# Long-Term Socialization with Humans Affects Human-Directed Behavior in Goats

**DOI:** 10.3390/ani10040578

**Published:** 2020-03-30

**Authors:** Vincenzo Mastellone, Anna Scandurra, Biagio D’Aniello, Christian Nawroth, Fiorella Saggese, Pasqualino Silvestre, Pietro Lombardi

**Affiliations:** 1 Department of Veterinary Medicine and Animal Productions, University of Naples Federico II, via Delpino 1, 80137 Naples, Italy; vincenzo.mastellone@unina.it (V.M.); pilombar@unina.it (P.L.); 2 Department of Biology, University of Naples Federico II, 80126 Naples, Italy; biagio.daniello@unina.it; 3 Institute of Behavioural Physiology, Leibniz Institute for Farm Animal Biology, Wilhelm-Stahl-Allee 2, 18196 Dummerstorf, Germany; nawroth@fbn-dummerstorf.de; 4 Lo Zoo di Napoli, Viale Kennedy 76, 80129 Naples, Italy; fiorella.saggese@lozoodinapoli.it (F.S.); pasquale.silvestre@lozoodinapoli.it (P.S.)

**Keywords:** cognitive test, domestication, evolution, goat behavior, heterospecific communication, impossible task

## Abstract

**Simple Summary:**

Goats are a useful model species to explore the effects of ontogenesis on the socio-cognitive abilities of domestic non-companion animals. The aim of this research was to study the behavioral response of goats with different socialization backgrounds to humans in the impossible task paradigm. Two groups of goats (high and low levels of socialization) were tested. Highly socialized goats interacted more with humans (the experimenter) during the test, while the low socialization group exhibited a higher level of interaction with the exit door.

**Abstract:**

Throughout their evolutionary history, humans have tried to domesticate a variety of wild terrestrial mammals, resulting in a limited number that has been successfully domesticated. Among these domesticated species, domestic goats (*Capra aegagrus hircus*) are a useful model species to study the effects of ontogenesis on the socio-cognitive abilities of domestic non-companion animals in their interactions with humans. To this end, the behavioral responses of two groups of goats with a different background of human socialization (high and low socialization) were compared in the impossible task test, an experimental paradigm aimed to study socio-cognitive skills and the tendency to interact with humans. Our results show that, when the task became impossible to solve, goats with a higher level of socialization interacted with the experimenter for a greater amount of time than subjects in the low socialization group, whereas the latter group exhibited increased door directed behavior. Overall, highly socialized goats made more social contact with humans compared to the other group in the impossible task paradigm.

## 1. Introduction

After shifting from a hunter-gatherer lifestyle to agricultural societies, humans started to domesticate different animal and plant species [1]. This process required rapid genetic change under anthropogenic-driven pressure, whereby some individuals of the species diverged from their wild ancestors [2,3]. Apart from morphological adaptiveness, psychological and behavioral changes allowing for the successful integration of the species in the new context are also expected.

Humans have tried to domesticate a variety of wild terrestrial mammals, most of which were successfully tamed; however, for several reasons, only about 10% were successfully domesticated [4]. Undoubtedly, dogs (*Canis lupus familiaris*) are among the most studied domestic mammals with respect to the effect of domestication on socio-cognitive skills when interacting with humans. Two theories aim to explain how dogs improved such abilities, i.e., the “Domestication Hypothesis” [5,6,7] and the “Two-Stage Hypothesis” [8,9,10], which are predicated on genetic and ontogenetic inputs, respectively. According to the latter, dogs become skilled in reading and responding to human messages after accepting people as social companions very early during their ontogeny, and continue to learn how to interact with them during their lives. Evidence of genetic determinants is based on studies demonstrating that dogs outperform similarly reared wolves in using a human pointing gesture to locate hidden food [11,12]. However, in some studies, the performance of wolves in these tasks did not appear to be worse compared to dogs [13,14,15]. Moreover, some authors denied the effect of ontogenesis on acquiring socio-cognitive skills in dogs ([16] but see also [17] for a rebuttal). On the other hand, under-socialized dogs show more difficulties comprehending human communicative signals with respect to pet dogs [18,19], although other research observed no differences according to socialization level [15]. Furthermore, a study on free-ranging dogs underlined the importance of learning from ontogenetic experience in the use of the human pointing gesture [20]. Certainly, genetic and ontogenetic contributions are both important factors underpinning the socio-cognitive skills in dogs [21,22], but their relative weight remains to be determined.

In contrast to dogs, goats have been bred for production traits rather than companionship. This makes them an interesting model species to study in terms of the effects of ontogenesis on the development of socio-cognitive abilities in domestic non-companion animals [23]. Although goats’ socio-cognitive skills have been the topic of recent research ([24] for a review), how ontogenetic factors affect these skills has rarely been studied. A better understanding of how goats interact with handlers, and how these interactions are affected by different degrees of socialization with humans, is also of relevance for improving the welfare of these animals. Indeed, increasing our knowledge in this area can help to improve handling procedures, and thus, decrease stress [25].

One experimental paradigm which aims to study socio-cognitive skills and, in particular, the tendency to interact with humans, is the so-called “impossible task” paradigm [11]. This task consists of several solvable trials in which a test subject learns to solve a simple challenge (e.g., opening the lid of a box) to obtain a reward. After that, the apparatus is locked, and the reward becomes inaccessible. The impossible task is similar to the problem-solving paradigm, but in the latter, a crucial element (i.e., violation of expectation) is missing. Previous research with this paradigm has shown that ontogenetic experiences are very important in shaping dogs’ socio-cognitive skills [26,27]. In particular, it was observed that the amount of social interaction with humans is strongly predictive of the tendency of dogs to interact with humans [28,29]. However, a study on Nigerian dwarf goats found no effect of short-term human handling on human-directed behavior [30]. In this research, an experimenter positively handled a group of goats, interacting for 30 min twice daily over two weeks. During this time, the goats received friendly talking, gentle touching, stroking, and hand feeding. Furthermore, the goats were also habituated to manage the experimental apparatus to retrieve food. This group of goats was then compared, in the impossible task paradigm, with goats only receiving standard husbandry care. The results showed no difference between the groups in their social interaction with the experimenter after three repetitions. Overall, the outcomes demonstrated that short-term human socialization was ineffective in increasing interactive behaviors toward humans in goats. Nevertheless, the authors discussed the possibility that the amount of human interaction in their study could have been insufficient to induce behavioral changes [30]. Furthermore, human socialization in Langbein et al. [30] was provided in adult age, whereas early socialization could also be important. Indeed, only early socialization allows wolves to accept humans as social companions, while older wolves are difficult to tame [31] and are not easy to handle [12], which holds also for feral dogs showing no evidence of socialization with humans [32].

Investigations on the effects of early socialization on human-directed behavior in goats are of interest because their offspring is precocial (in contrast to canids), which makes it possible for different forms of contact to occur with humans during early life stages. We compared the behavioral responses of two groups of goats with a different socialization background in the impossible task paradigm. The aim of the present research was to verify whether early and/or long-term socialization affect the inclination of goats to interact with humans when they encounter a problem that they cannot solve by themselves. We predicted that highly socialized goats should exhibit more human-directed behaviors than goats that have not received this level of socialization.

## 2. Materials and Methods 

### 2.1. Subjects and Housing 

Thirty Tibetan Plateau goats from the zoo of Naples, Italy, participated in this experiment. It was not possible to test all the goats because some did not meet the criteria to be admitted in the impossible task procedure (see below). Hence, nine goats in the low socialization group (e.g., control, 2 males and 7 females) and ten in the high socialization group (e.g., socialized, 2 males and 8 females) were finally tested. The experiment was performed according to the Animal Welfare and Good Clinical Practice (Directive 2010/63/EU). For all animals, health status was assessed by clinical examination before the beginning of the experiment. Sixteen goats belonged to a standard husbandry group (control; mean age ± SD: 32.8 ± 29.08 months) and fourteen to a group involved in education programs (individuals had long-term social contact with children and adults entering the enclosure almost daily): a socialized group (mean age ± SD: 19 ± 14.97 months). Although the control group included older goats, no statistical differences on the goats’ age were found. All goats had been similarly reared by the caregivers of the zoo since birth, and were hosted in two different irregular enclosures of about 100 m^2^. Visitors of the zoo could enter the socialized goats’ enclosure during their visit to the zoo in the presence of the caregivers, whereas they were not allowed to enter the control goats’ enclosure.

### 2.2. Experimental Procedure

The procedure consisted of two stages: habituation and testing. Both took place within two rectangular wooden sheds with an area of about 8 m^2^ and a height of about 2 m inside the fence. The sheds were usually used as shelter by the goats and were now designated as the test arena for this experiment.

#### 2.2.1. Habituation

The aim of this stage was to allow the goats of both groups to familiarize themselves with the experimental apparatus in the test arena and the experimenter. The habituation consisted of four different phases in which the experimenter drew the attention of the goats by giving them food (raw pasta). In total, the habituation procedure lasted 24 non-consecutive days, depending on the weather conditions (i.e., no habituation took place during days of rain or excessive wind [33].

Phase 1: all goats spent 30 min a day, for two days, inside the test arena of their enclosure with the experimenter.

Phase 2: the procedure was similar to phase 1, but only two goats entered the test arena each time with the experimenter. Overall, the experimenter spent 1 h in each test arena. This phase lasted for 2 days.

Phase 3: the experimenter entered the test arena with only one goat at a time, isolating the goat from the rest of the group. The procedure was repeated for 15 days, progressively increasing the time spent in the test arena. For each goat, the time spent with the experimenter was from about 30 s in the first days up to 2 min at the end of the phase. 

Phase 4: the procedure was similar to the previous phase, but the experimenter had previously positioned the empty experimental apparatus (see below for a detailed description) inside the test arena. This phase lasted 4 days and was repeated once after 2 weeks. On the first day, the experimenter presented the experimental apparatus with only the wooden base and the lid screwed on. On the second day, the upside-down plastic container was added, to accustom the goats to the configuration in which they were going to see the apparatus during the test phase.

Overall, individual goats entered the test arena once a day. 

#### 2.2.2. Test

The experimental apparatus consisted of a plastic food container (11.5 × 11.5 cm) positioned upside-down on a rectangular wooden base (38 × 15 cm). The lid of the food container was fixed on the wooden base and the food container was either loosely placed on the lid during the solvable trials or locked onto the lid during the impossible trial. 

The procedure involved only one experimenter and consisted of three consecutive solvable trials and a subsequent impossible trial. During the solvable trials, the experimenter placed some pieces of food on the lid and covered it with the food container, luring the goats toward the apparatus with a piece of pasta in hand, if necessary. The task could be solved by moving away the container using the muzzle or the hoof. The goats that failed to pass three consecutive solvable trials within a total time of two minutes underwent the test again for a maximum of three sessions on three different days. The goats that, despite the three sessions, failed to solve the three trials, were not subjected to the impossible trial and were excluded from the final sample. During the impossible trial and after locking the food container, the researcher remained in a stationary position about 30 cm from the wooden board of the apparatus, alternating randomly between the right and the left side, staring straight ahead and ignoring the goat for the duration of the test trial (60 s).

### 2.3. Data Analysis 

All tests were video recorded with a Camcorder (HDR-PJ260VE; Sony, Tokyo, Japan) provided with a wide-angle lens, positioned in a high corner of the test arena. The behavior of the goats in the impossible trial (Table 1) was coded by a trained researcher using Solomon Coder^®^ beta 19.08.02 (ELTE TTK, Budapest, Hungary) to analyze the videos. A second independent researcher randomly coded 25% of the final sample for interobserver reliability, and a high level of agreement (from 95% to 99%) was found for all the behaviors in all ethological parameters. 

For all behaviors, frequency (i.e., the number of times), duration (i.e., the time expressed in seconds) and latency (i.e., the time in seconds from start of the trial to the first occurrence of the behavior) were analyzed. Most of the data were not normally distributed and the sample size was limited; thus, we used a nonparametric statistical approach by Mann-Whitney pairwise comparison [34]. The statistical analysis was run by using the Past software^®^ [35].

## 3. Results

Ten goats in the control group and nine in the socialized group reached the criteria of three consecutive trials. Six goats in the control group reached the criteria in the first session, one in the second, and two in the third, while nine goats in the socialized group reached the criteria in the first session and one in the second. Experimenter-directed behaviors were only occasionally observed in some subjects during the solvable phases but were not included in further statistical analyses. Descriptive statistics for the impossible trial are reported in Table 2.

**Figure 1:**
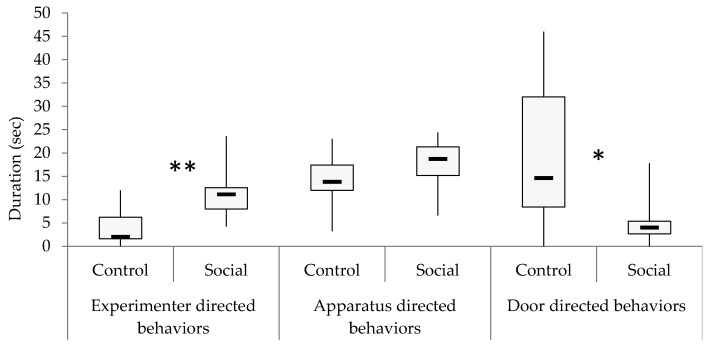
The duration (in seconds) of Experimenter/Apparatus/Door directed behaviors in the control and socialized groups. Black rectangles: medians; boxes: from 25 to 75% quartiles; thin vertical lines: minimum and maximum values. * *p* < 0.05, ** *p* < 0.01.

The goats in the socialized group showed a higher median duration of experimenter-directed behavior than the control group (U = 11; z = −2.74; *p* = 0.006) (Figure 1). This difference appeared as a tendency for frequency (U = 21.5; z = −1.89; *p* = 0.06). Latency for experimenter-directed behavior was shorter in the socialized group, although also only as a tendency (U = 22; z = −1.84; *p* = 0.07). When considering single behaviors, it appeared that the medians of visual, tactile approaches, and going toward the experimenter, were always higher in the socialized group, both in duration and frequency, while the latencies were shorter (see Table 2). Statistical differences between group medians appeared for the duration (U = 16; z = −2.39; *p* = 0.02) and the frequency (U = 19; z = −2.16; *p* = 0.03) of tactile approaches. Significant differences were found for the duration of door-directed behaviors (U = 16.5; z = −2.29; *p* = 0.02), with the control group showed longer durations of displaying behavior towards the door (Figure 1); no statistical differences were found for frequency and latency. Tactile approach to the door appeared higher in duration (U = 24; z = −1.93; *p* = 0.05) and frequency (U = 24; z = −1.94; *p* = 0.05) in the control group. No other differences in duration, frequency, and latency of the single door-directed behaviors appeared. There were neither statistical differences for the apparatus-directed behaviors category, nor for all behaviors examined and reported in Table 2.

## 4. Discussion

In this study, we provide a comparison between goats that are highly socialized from early life and goats that experienced standard husbandry (control), using the impossible task paradigm. We found that goats with high socialization levels interacted with the experimenter more often compared to the control group when the task became impossible to solve. Interestingly, dogs seem to interact with humans prevalently through gazing behavior in this paradigm [36], while the behavior of our goats suggests that physical contact seems very important. Since Miklósi et al. [37], gazing behavior (also expressed as so-called ‘looking back’) in the impossible task paradigm has been interpreted as a request for help to solve the task in dogs. However, a recent paper questioned such a hypothesis [38]. Therefore, it is not clear whether the human-directed behaviors shown by our goats can be interpreted as requests for help to solve the task. It could be a form of begging behavior once the subjects realized that the food had been rendered inaccessible within the apparatus. In the current study, there was only one human present who also baited the reward. As the human is doing both, i.e., baiting and being available, begging could be a more likely explanation than asking for help. Tactile interaction is also used in intraspecific communication in goats (e.g., grooming, fighting). The behavior displayed in our study (tactile approach) might be considered a socio-positive (or at least neutral) behavior, because no antagonistic events occurred. However, in a food (and potential food competition)-context such as the impossible task, goats do not rely on such subtle tactile interactions when dealing with conspecifics. In any case, it is evident from our data that socialized goats appear to be more interactive with humans. The other behavior that differed between the two groups was the interaction with the door, which was more likely in the control group. This behavior indicates the inclination to move away rather than interact with the human experimenter.

Goats socialized for two weeks did not show such differences [30]. It is possible that the longer period of socialization of our goats could explain this discrepancy with the previous results, but also, that early socialization could have been a determinant. Our data do not allow us to disentangle the relative effect of the two factors. A correlational analysis between age and human-directed behavior may have been helpful in such a context. However, our sample size was too limited to reach robust conclusions. In any case, the results of the present paper provide strong support that ontogenetic processes, such as socialization with humans, can affect the socio-cognitive skills of goats.

In a previous study, it was hypothesized that horses (and probably domestic animals not bred for companionship in general) show decreased performance compared to dogs in their use of human-given cues in an object-choice task paradigm [39]. The authors suggested that simpler mechanisms underpin horses’ responses, such as stimulus enhancement, rather than comprehending the communicative nature of human signals [39,40]. However, dogs living in human families have, on average, more opportunities to socialize with humans than the horses and goats used in similar studies, which could explain the improved performance observed in dogs. In addition, species-specific adaptations in the different taxa, such as different feeding strategies and social group structures, might explain some, if not most, of the variance found. Whereas the impossible task paradigm measures the tendency of animals to interact with humans, the object-choice task paradigm attempts to anticipate an animal’s response to human communicative cues. It would be of interest to know whether long-term socialization with humans also improves to performance of goats in an object-choice task [40,41], as has been shown for dogs [28]. 

Although our results demonstrate behavioral differences depending on goat’s socialization levels with humans, there are limitations in the interpretation of our data. Of particular interest in such a context is the gazing behavior that increases with human socialization in dogs [28]; this behavior has also been shown by goats when interacting with humans in previous studies using the impossible task paradigm [30,42]. Our restricted sample size, and the associated decrease in statistical power in our study, might have prevented us from finding stronger support for the effects of more subtle behavioral parameters. However, we could not increase our sample size due to difficulties in acquiring more subjects which had been exposed to similar rearing backgrounds. On the other hand, we also encountered difficulties in testing these goats. Indeed, we had to exclude several subjects since they failed the experimental procedure in the solvable phase after three sessions on different days.

From an applied perspective, these findings may also be of relevance to the welfare of goats in farm and zoo settings, because individual differences in socio-cognitive capacities can affect how goats adapt to human handling [24]. Together with several environmental factors, suboptimal human–animal interactions have already been identified as an important element affecting goats’ welfare [43]. Early and increased social behavior with humans may help to reduce the stress caused by handling procedures, thus improving human–animal interactions in the long-term. 

## Figures and Tables

**Table 1:** Ethogram applied for the study. All behaviors are mutually exclusive, except for the bleating.

Behaviors	Definitions	Targets
Visual approach	From a stationary position, the goat turns/lifts head towards the target, without approach	Experimenter Apparatus Door
Tactile approach	The goat establishes physical contact with the target, e.g., rubbing, nosing, pawing a hand or leg or jumping up	
Go towards	The goat moves in the direction of the target	
Stress behaviors	Locomotion (move around the test arena without precise orientation); bleating	

**Table 2:** Mean ± standard deviation of behaviors coded during the impossible trial. * indicates significant differences (*p* < 0.05) between the control group and socialized group.

Categories	Behaviors	Group	Frequency	Duration (s)	Latency (s)
Experimenter-directed behaviors	Visual approach	Control	4.11 ± 2.76	2.87 ± 2.40	16.78 ± 21.10
		Social	5.70 ± 2.36	4.64 ± 1.97	4.28 ± 6.37
	Tactile approach	Control	0.56 ± 0.73 *	0.76 ± 1.11 *	40.96 ± 22.90
		Social	1.90 ± 1.37 *	5.76 ± 6.11 *	23.68 ± 22.90
	Go towards	Control	0.67 ± 1	0.71 ± 1.30	43.96 ± 21
		Social	1.70 ± 1.42	1.52 ± 1.16	32.86 ± 20.90
Apparatus-directed behaviors	Visual approach	Control	2.22 ± 1.48	1.87 ± 2.05	27.96 ± 25.3
		Social	3.60 ± 2.12	2.50 ± 2.72	12.86 ± 18
	Tactile approach	Control	2.89 ± 1.50	10.44 ± 6.50	0.89 ± 2.19
		Social	4.40 ± 2.10	13.60 ± 4.52	1.48 ± 4.28
	Go towards	Control	0.67 ± 1	1.18 ± 2.01	42.42 ± 24.90
		Social	1.40 ± 1.58	1.60 ± 1.54	40.56 ± 23.80
Door-directed behaviors	Visual approach	Control	3.56 ± 3.28	5.24 ± 5.83	18.73 ± 23.70
		Social	3.40 ± 2.72	3.28 ± 3.31	23.36 ± 16.90
	Tactile approach	Control	2.11 ± 2.8	9.89 ± 11.60	39.60 ± 22.90
		Social	0.20 ± 0.40	0.54 ± 1.34	53.02 ± 14.90
	Go towards	Control	2.33 ± 2.4	3.96 ± 3.12	27.40 ± 22.40
		Social	1.50 ± 1.27	1.74 ± 1.64	37.28 ± 16.60
